# Comparative Analysis of Gut Microbiomes in Parasitic Roundworms Reveals Phylogeny‐Associated Community Structure and Functional Adaptation

**DOI:** 10.1155/tbed/2764696

**Published:** 2026-04-30

**Authors:** Xinyi Fan, Xuan Zhou, Lidan Wang, Xinhui Zhang, Yuanyuan Shen, Yating Xiao, Hui Wang, Lei Deng, Yue Xie

**Affiliations:** ^1^ College of Veterinary Medicine, Sichuan Agricultural University, Chengdu, 611130, China, sicau.edu.cn; ^2^ Shanghai Veterinary Research Institute Chinese Academy of Agricultural Sciences, Shanghai, 200241, China; ^3^ Sichuan Provincial Forestry and Grassland Key Laboratory of Biodiversity Conservation and Sustainable Community Development in Giant Panda National Park, Chengdu Normal University, Chengdu, 611130, China, cdnu.edu.cn; ^4^ College of Animal Science and Technology, Sichuan Agricultural University, Chengdu, 611130, China, sicau.edu.cn; ^5^ Agricultural Animal Diseases and Veterinary Public Health Key Laboratory of Sichuan Province, Sichuan Agricultural University, Chengdu, 611130, Sichuan, China, sicau.edu.cn

**Keywords:** Ascaridida, functional adaptation, gut microbiota, phylosymbiosis

## Abstract

Roundworm nematodes are globally distributed zoonotic parasites that inhabit the intestinal tract of various mammals. Although these parasites reside in the host’s guts, their own intestinal ecosystems remain poorly understood. Recent evidence suggests that helminths may harbor distinct gut microbiomes that contribute to their physiology and host interactions, yet cross‐species comparisons are lacking. Here, we performed full‐length 16S rRNA sequencing to characterize and compare the gut microbiomes of four major roundworm species—*Ascaris suum (As)*, *Baylisascaris schroederi (Bs)*, *Toxocara cati (Tc)*, and *Toxocara vitulorum (Tv)*. Across 38 individual worms, we identified 359 bacterial taxa dominated by Enterobacteriaceae, with *Escherichia coli*, *Salmonella enterica*, and *Klebsiella pneumoniae* forming a conserved core community. Despite this compositional similarity, beta‐diversity and hierarchical clustering analyses revealed that microbial community structure was primarily determined by parasite phylogeny and roundworm sex, not host diet. Functional prediction using PICRUSt2 indicated clear species‐specific enrichment in metabolic pathways, such as carbohydrate metabolism in *Bs* and xenobiotic metabolism in *As*, reflecting adaptive divergence of microbial functions. Collectively, these findings demonstrated that roundworm gut microbiomes exhibited taxonomic conservation but functional specialization, shaped by the evolutionary history of the parasites themselves. This study established a conceptual framework viewing the parasite as the primary host of its microbiome and provided new insights into the co‐evolutionary relationships between helminths and their symbiotic bacteria.

## 1. Introduction

Roundworm nematodes (order Ascaridida) represent one of the most ubiquitous groups of zoonotic helminths, infecting a broad range of mammalian hosts and causing substantial veterinary and public health burdens [1–3]. Three major genera—*Ascaris*, *Toxocara*, and *Baylisascaris*—distributed across two distinct subfamilies, account for much of this morbidity and socioeconomic impact [4–6]. In definitive hosts, these parasites typically undergo a hepato‐tracheal larval migration, and some species additionally achieve vertical transmission via the placenta or milk [2, 7]. In intermediate or paratenic hosts, however, larvae may migrate to the viscera (visceral larva migrans, VLM), eye (ocular larva migrans, OLM), or nervous system (neural larva migrans, NLM), leading to severe clinical manifestations including epilepsy and neuropsychiatric disorders [7, 8]. Human infections are commonly associated with *Ascaris lumbricoides* (ascariasis), *Toxocara canis* (toxocariasis), and *Baylisascaris procyonis* (baylisascariasis) with high seroprevalence among school‐aged children in tropical and subtropical regions and in disadvantaged communities in some countries [6, 9–11].

As with all gastrointestinal nematodes, these roundworms inhabit the intestinal lumens of their definitive hosts. Traditionally, their biological impact has been framed in terms of malnutrition, impaired growth, cognitive stunting, and immune dysfunction [2, 3]. However, increased evidence suggested that these effects extended beyond direct parasite‐host interactions and were strongly influenced by the host intestinal microbiota. For instance, *Ascaris*‐derived antimicrobial peptides (e.g., cecropins and lysozymes) can interfere with bacterial biofilm formation, thus limiting the proliferation of pathogenic *Escherichia coli* within the intestinal lumen of the host [12]. *Ascaris*‐producing C‐type lectins can also specifically agglutinate pathogens like *Salmonella* via calcium‐dependent mechanisms to prevent epithelial invasion [13]. In addition, the immune responses elicited by *Ascaris* infections profoundly reshaped the gut’s ecological balance, suppressing the growth of specific commensal bacterial species while simultaneously fostering the growth of others, such as *Vibrio cholerae* [14]. While substantial research has focused on how roundworms influence the host gut microbiome, significantly less attention has been paid to the microbiome within the parasite itself so far [15, 16].

Nevertheless, several studies have shown that the gut microbiome of parasitic nematodes, including roundworm species, plays a crucial role in their biology and survival [14, 17–19]. For example, White et al. [20] demonstrated that the mouse whipworm *Trichuris muris* relied on its intestinal bacterium *Bacteroides thetaiotaomicron* to establish infection and maintained long‐term survival in the host. Conversely, antibiotic treatment significantly reduced the parasite’s fitness, underscoring the essential role of the parasite‐resident intestinal microbiota for whipworm parasitism. Moreover, endosymbiotic *Wolbachia* have also been exploited for the treatment of human filariasis. These bacteria are essential for worm development, larval molting, and embryogenesis within *Brugia* spp. and *Onchocerca* spp. Therefore, reducing *Wolbachia* abundance by disrupting glycolysis or pyruvate availability in the host significantly impairs parasite reproduction and survival [21–23]. For roundworm nematodes, two microbiome studies on the horse *Parascaris* spp. and pig *Ascaris suum* recently confirmed that both species harbored a distinct bacterial community in their guts, composed of Firmicutes, Proteobacteria, and other taxa that were under‐represented in the host microbiomes [24, 25]. It is clear that the composition of the parasite’s microbiome is influenced by both the host environment and the parasite’s own metabolic processes [17, 24–26]. However, the specific composition and functional role of the microbiome within different roundworm species remain poorly understood.

To bridge this knowledge gap, we performed full‐length 16S rRNA gene sequencing on four representative roundworm species from the three major genera of concern: *A. suum (As*), *Baylisascaris schroederi (Bs)*, *Toxocara cati (Tc)*, and *Toxocara vitulorum (Tv)* (Figure 1a). By analyzing these distinct species, we aimed to characterize their internal microbiota and disentangle the ecological and evolutionary forces driving community assembly. Specifically, this study addressed three fundamental questions: (i) Do roundworm gut microbiomes share a core microbial composition across divergent species? (ii) Is the variation in microbial communities primarily constrained by parasite phylogeny and sex, or is it driven by host‐related factors such as diet? (iii) Do differences in taxonomic composition correspond to distinct functional potentials? By systematically addressing these questions, this work establishes a conceptual framework for viewing parasites not merely as pathogens but as autonomous hosts of their own microbial ecosystems, offering new insights into nematode biology and co‐evolution.

Figure 1Experimental workflow and taxonomic characterization of the roundworm gut microbiome. (a) Schematic overview of the experimental design encompassing the collection of four roundworm species (*A. suum* [As], *B. schroederi* [Bs], *T. cati* [Tc], and *T. vitulorum* [Tv]) from their respective definitive hosts, sample processing, full‐length 16S rRNA gene sequencing using the Oxford Nanopore platform, and the subsequent bioinformatic pipeline for taxonomic and functional profiling. (b) Taxonomic composition at the family (top) and species (bottom) levels. The stacked bar plots illustrate that the roundworm gut is overwhelmingly dominated by the family Enterobacteriaceae and a conserved core community consisting of a few dominant species (e.g., *E. coli*, *S. enterica*, and *E. marmotae*), suggesting a highly specialized and streamlined microbial ecosystem across all four roundworm species.

**Figure:**
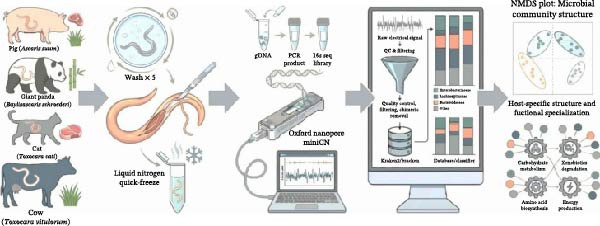
(a)

**Figure:**
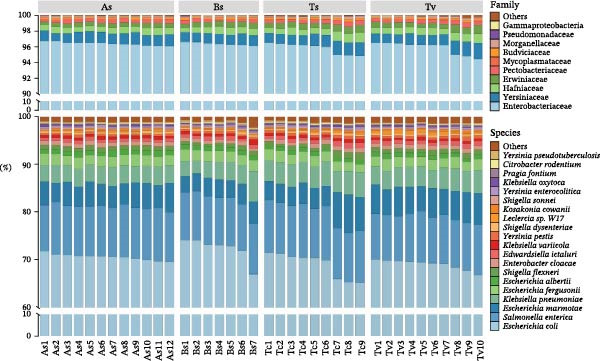
(b)

## 2. Methods

### 2.1. Ethical Statement

This study was approved by the Animal Ethics Committee of Sichuan Agricultural University, China (Approval Number SYXK 2014‐187) and the Wildlife Management and Animal Welfare Committee of China. All procedures involving animals adhered to the Guide for the Care and Use of Laboratory Animals (National Research Council, MD, USA) and followed the ARRIVE guidelines.

### 2.2. Parasite Collection and Processing

Roundworm nematodes were collected from naturally infected definitive hosts during routine clinical or post‐mortem procedures. Four species were included: *As* (*n* = 12) from sows at a local abattoir (Chengdu, China), *Bs* (*n* = 7) from an injured and rescued giant panda (Dujiangyan, China), *Tc* (*n* = 9) from cats housed in an animal shelter (Qionglai, China), and *Tv* (*n* = 10) from a Simmental calf on a dairy farm (Yaan, China). Each individual worm was treated as one biological replicate. To minimize contamination from the host luminal microbiota and ensure accurate profiling of the internal parasite microbiome, a rigorous surface sterilization protocol was employed, as described elsewhere [18, 19]. All nematodes were washed five times with warm RPMI‐1640 media containing antibiotics, incubated for 1 h at 37°C, and subjected to five additional washes. Following this, the complete intestine of each worm was aseptically dissected. The entire intestinal tract was collected as a single sample (each weighing >200 mg), immediately flash‐frozen in liquid nitrogen, and stored at −80°C until DNA extraction.

### 2.3. DNA Extraction and 16S rRNA Gene Sequencing

Genomic DNA (gDNA) was extracted from each intestinal sample using the ZymoBIOMICS DNA Microprep Kit (Zymo Research, Irvine, CA, USA) following the manufacturer’s protocol. An extraction blank was processed simultaneously to monitor for reagent contamination. DNA integrity was verified via 0.8% agarose gel electrophoresis, and concentration was quantified using the PicoGreen dye method on a Tecan F200 microplate reader. Afterwards, the full‐length bacterial 16S rRNA gene was amplified using indexed primers 8F and 1492R [27]. PCR reactions (25 μL) contained 5 μL of 5× MegaFi Buffer, 0.5 μL of 10 mM dNTPs, 1 μL of each primer (10 μM), 0.5 μL of MegaFi DNA Polymerase (ABM, Vancouver, Canada), 2 μL of template DNA (20 ng), and 15 μL of nuclease‐free water. Thermal cycling was performed on an Applied Biosystems 9700 with the following profile: initial denaturation at 98°C for 30 s; 30 cycles of 98°C for 5 s, 54°C for 15 s, and 72°C for 45 s; and a final extension at 72°C for 2 min. A negative control was included in each run, and samples were amplified in technical triplicate. Target amplicons (~1.5 kb) were confirmed by 1% agarose gel electrophoresis, excised, and purified using the Zymoclean Gel DNA Recovery Kit (Zymo Research). Purified amplicons were quantified, pooled in equimolar amounts, and prepared for sequencing. End repair and dA‐tailing were performed using NEBNext reagents (New England Biolabs, USA), followed by adapter ligation using the Ligation Sequencing Kit (SQK‐LSK109; Oxford Nanopore Technologies). Sequencing was carried out on a GridION X5 platform using a MinION Flow Cell (R9.4.1; FLO‐MIN106D). The flow cell was primed following the ONT Flow Cell Priming Kit (EXP‐FLP002) protocol and controlled via MinKNOW software. A 75 μL loading mix comprising 37.5 μL SQB, 26.5 μL LB and 11 μL of the final library was loaded onto the flow cell, and sequencing was run for 48 h.

### 2.4. Bioinformatic Analysis and Taxonomic Assignment

Raw Nanopore signal data were basecalled using Guppy v6.5.7 to generate FASTQ files with per‐read quality scores. Reads with a mean quality score < Q7 (80% accuracy, consistent with ONT 16S recommendations) were removed using NanoFilt v2.8.0 [28]. Chimeric sequences were identified and removed with the UCHIME algorithm using the Gold database as a reference (https://www.drive5.com/uchime/uchime_download.html) [29]. Taxonomic classification was performed using the Kraken2 v2.17.1 engine (https://github.com/DerrickWood/kraken2/releases), with species abundance re‐estimated and corrected using Bracken v3.1 (https://github.com/jenniferlu717/Bracken/releases). Samples yielding fewer than 5000 total reads were excluded from downstream analysis. For this study, each species‐level taxon assigned by the Kraken2/Bracken pipeline was treated as an operational taxonomic unit (OTU) in the sense of a species‐level aggregated unit, distinct from traditional OTUs defined by sequence‐similarity clustering. All subsequent references to OTUs referred to these species‐level taxonomic units [30, 31].

### 2.5. Statistical Analysis of Community Diversity

All statistical analyses were conducted in the R environment v4.3.2. Comparative analyses were stratified across four dimensions: (1) parasite species (As, Bs, Tc, and Tv); (2) parasite genus (*Ascaris*, *Baylisascaris*, and *Toxocara*); (3) host diet (carnivorous, herbivorous, and omnivorous); (4) parasite sex (female and male). To normalize sequencing depth, samples were rarefied to 15,000 reads using the GUniFrac package (https://github.com/jchen1981/GUniFrac). Shannon rarefaction and rank‐abundance curves were generated to assess coverage. Alpha diversity indices (Chao1 for richness and Simpson for evenness) were calculated using the Microbiome package (https://github.com/microbiome/microbiome). Differences between groups were evaluated using the Kruskal–Wallis test, followed by pairwise Wilcoxon rank‐sum tests with Holm–Bonferroni correction for multiple comparisons. Beta diversity was quantified using Bray–Curtis dissimilarity. Non‐metric multidimensional scaling (NMDS) was applied to the Bray–Curtis distance matrix for ordination and visualization, including 95% confidence ellipses for group centroids. Group‐level differences in community composition were tested using a nested PERMANOVA design (adonis2, formula: ~genus/species + sex, 999 permutations), where species was nested within Genus to account for the hierarchical taxonomic structure of the roundworms. Additionally, homogeneity of multivariate dispersion among groups was evaluated using betadisper followed by permutest (999 permutations). All beta diversity analyses were performed with the vegan package v2.7‐2 (https://cran.r-project.org/web/packages/vegan/index.html).

### 2.6. Functional Prediction and Network Analysis

The functional potential of the microbiome was predicted from 16S rRNA data using PICRUSt2 v2.6.3 (https://github.com/picrust/picrust2), with results aggregated to KEGG Pathway Level 2. Linear discriminant analysis Effect Size (LEfSe) was employed to identify robust functional biomarkers driving differences among roundworm species (LDA score threshold >2.0). To explore ecological interactions, co‐occurrence networks were constructed based on significant correlations between bacterial taxa and predicted functional pathways (Spearman’s *ρ* > 0.6, *p* < 0.05). Data manipulation and visualization were performed using dplyr, tidyr, ggplot2, and ggpubr. Where necessary, the identities of significant taxonomic units were manually verified by alignment against the NCBI nr database using BLAST to ensure classification accuracy.

## 3. Results

### 3.1. Sequencing Overview and the Core Microbiome

We characterized the gut microbiota of 38 roundworms representing four species—As, Bs, Tc, and Tv—yielding a total of 1,005,685 high‐quality reads (Q ≥ 7). The dataset averaged 22,847 ± 4251 reads per sample, providing sufficient sequencing depth as evidenced by the clear plateaus of Shannon rarefaction curves (Supporting Information 1: Figure S1a) and the steep heads and long tails of rank‐abundance curves (Supporting Information 1: Figure S1b). Taxonomically, the dataset resolved into 359 OTUs spanning 7 phyla and ~215 species. The number of OTUs per roundworm species was similar in magnitude (Tc: 163, As: 160, Bs: 158, and Tv: 165), suggesting their comparable richness and detection depths.

Despite the host and species diversity, the roundworm gut microbiomes exhibited a striking “core” consistency (Figure 1b). The community was overwhelmingly dominated by the phylum Proteobacteria, specifically the family Enterobacteriaceae. The top five most abundant species—*Escherichia coli*, *Salmonella enterica*, *Escherichia marmotae*, *Klebsiella pneumoniae*, and *Shigella flexneri*—collectively accounted for a median relative abundance of 94.2% (ranging 89.1%–97.8%). This extreme dominance (median 94.2%) suggested that the roundworm gut environment exerted strong selective pressure favoring facultative anaerobes with versatile substrate utilization capabilities.

### 3.2. Community Diversity and Drivers of Assembly

To determine the factors driving microbiome assembly, we assessed alpha and beta diversity using rarefied data (15,000 reads/sample) at the species, genus, parasite sex, and host diet levels (Figure 2). Venn analysis confirmed the gut microbiome core similarity among the four roundworm species, which shared a 74.88% intersection‐to‐union ratio of species‐level OTUs, differing primarily in the low‐abundance “rare biosphere” rather than the dominant membership (Figure 2a). Alpha diversity was reflected by the Chao1 and Simpson index. Species richness (Chao1 index) was comparable across all groups, while community evenness (Simpson index) varied significantly among different roundworms at the species and genera level (Figure 2b,e). Specifically, the Bs group exhibited significantly lower evenness compared to the other three species, reflecting a more skewed abundance distribution (Figure 2b). Beta diversity analysis (NMDS based on Bray–Curtis dissimilarity) revealed that the parasite taxonomy at the species and genus levels was the primary determinant of community structure (Figure 2c,f). It appeared that samples were clearly separated by parasite species, but less pronounced when samples were grouped by genus (Figure 2c,f). In contrast, clusters by worm sex (female and male) and by host dietary category (carnivore, herbivore, and omnivore) exhibited substantial overlap with only weak visual separation (Figure 2i,l). The PERMANOVA analysis confirmed that the parasite genus (*R* ^2^ = 0.256, *p* = 0.00001) and species nested within the genus (*R* ^2^ = 0.069, *p* = 0.003) were the strongest explanatory variables. Additionally, the worm sex also had a statistically significant but small effect (*R* ^2^ = 0.067, *p* = 0.008) (Table 1). Moreover, the host diet explained less variation and contributed minimally relative to parasite taxonomy.

Figure 2Microbial community structure is primarily driven by parasite taxonomy rather than worm sex or host diet. (a–c) Species‐level comparison of the four roundworms. (a) Venn diagram showing shared and unique OTUs among As, Bs, Tc, and Tv. (b) Boxplots of alpha diversity (Chao1 richness and Simpson evenness); significant pairwise differences are indicated by asterisks. (c) NMDS ordination illustrating separation of communities by parasite species (95% confidence ellipses; stress value shown). (d–f) Genus‐level comparison of the four roundworms. (d) Venn diagram of shared and unique OTUs among the genera *Ascaris*, *Baylisascaris*, and *Toxocara*. (e) Boxplots of alpha diversity indices as in (b). (f) NMDS ordination showing separation by parasite genera. (g‐i) Effect of the roundworm sexes. (g) Venn diagram showing extensive OTU overlap between male (M) and female (F) roundworms. (h) Boxplots of alpha diversity showing no significant differences between sexes. (i) NMDS ordination showing that group centroids are close and confidence ellipses largely overlap, consistent with only modest differences in overall community structure. (j–l) Effect of the roundworm host diets. (j) Venn diagram of shared and unique OTUs among worms from carnivorous, herbivorous, and omnivorous hosts. (k) Boxplots of alpha diversity showing no significant differences among dietary categories. (l) NMDS ordination showing some separation, particularly for worms from herbivorous hosts, with stress value indicating good fit. Alpha diversity *p*‐values were obtained by Wilcoxon rank‐sum tests with Holm–Bonferroni correction;  ^∗∗^ *p* < 0.01,  ^∗∗∗^ *p* < 0.001. NMDS ordinations are based on Bray–Curtis dissimilarity.

**Figure:**
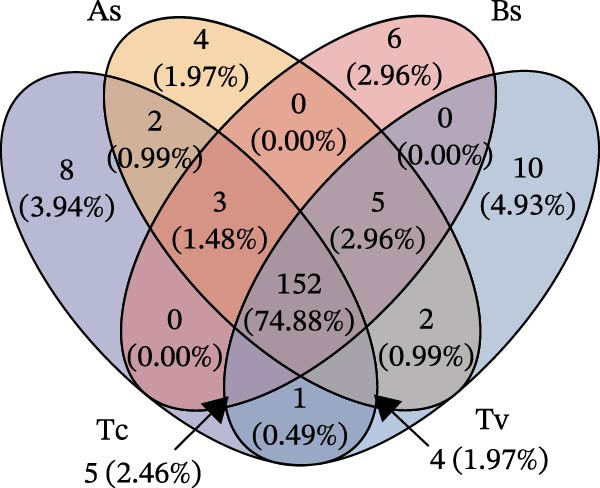
(a)

**Figure:**
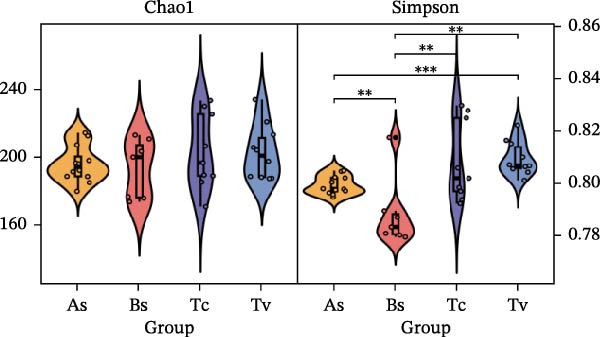
(b)

**Figure:**
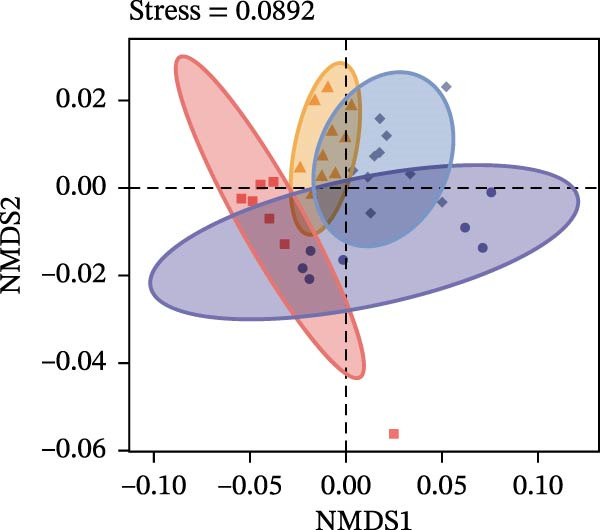
(c)

**Figure:**
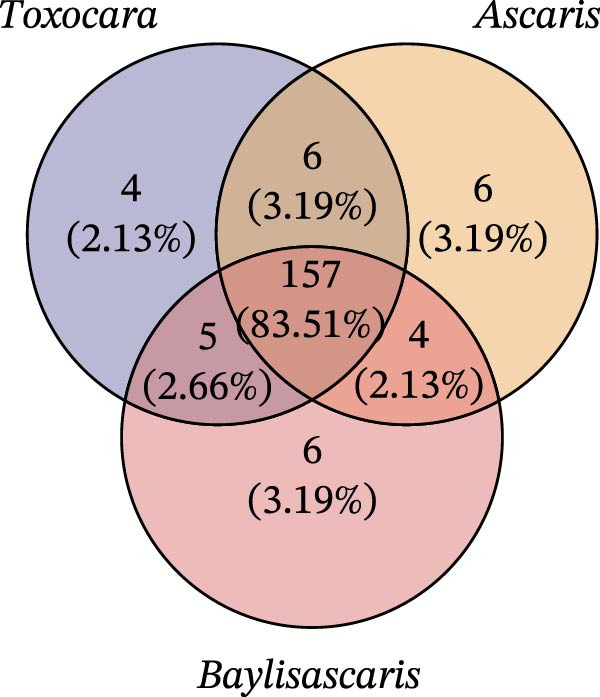
(d)

**Figure:**
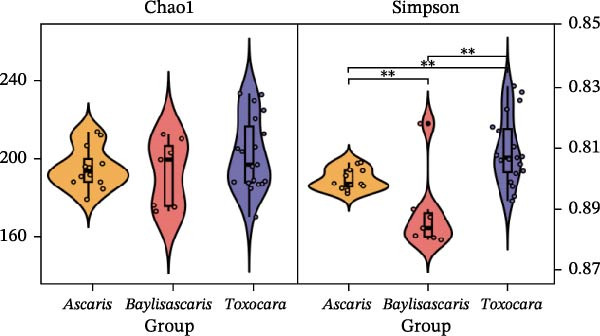
(e)

**Figure:**
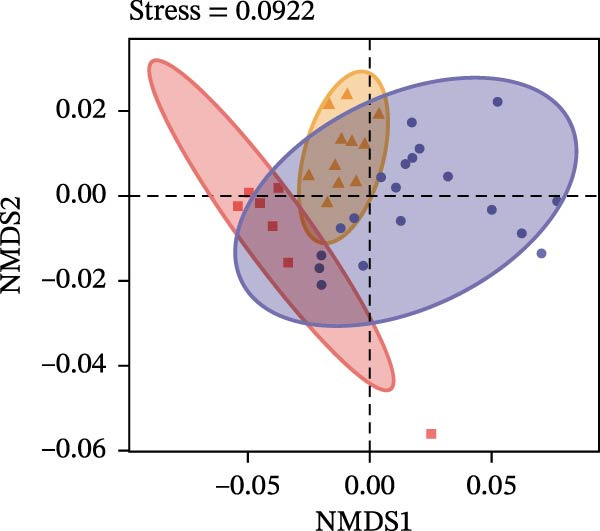
(f)

**Figure:**
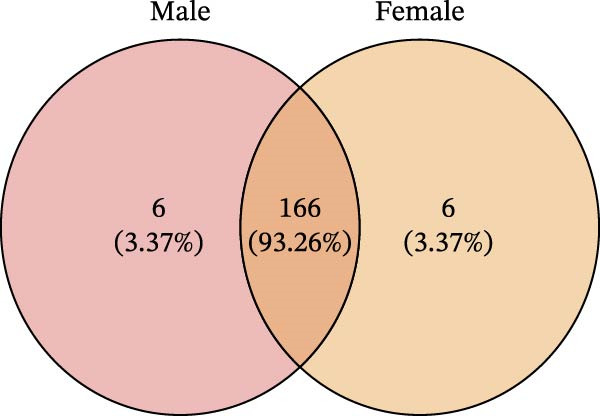
(g)

**Figure:**
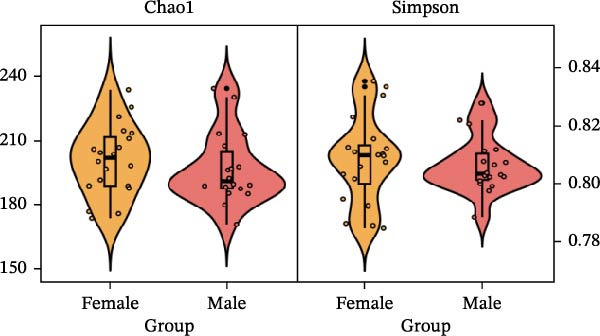
(h)

**Figure:**
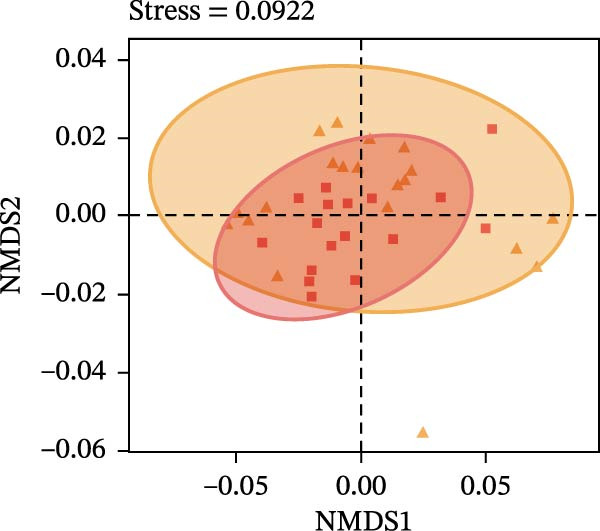
(i)

**Figure:**
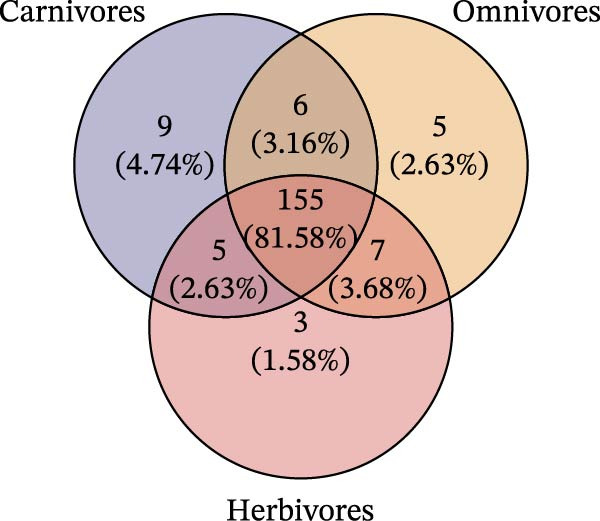
(j)

**Figure:**
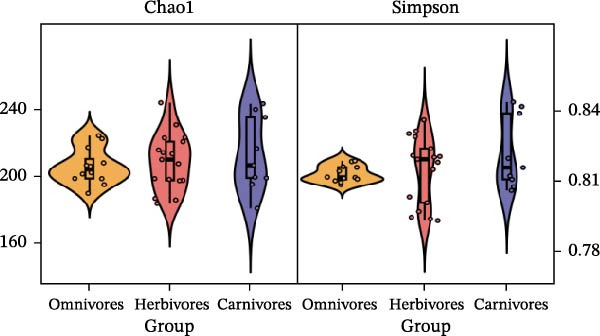
(k)

**Figure:**
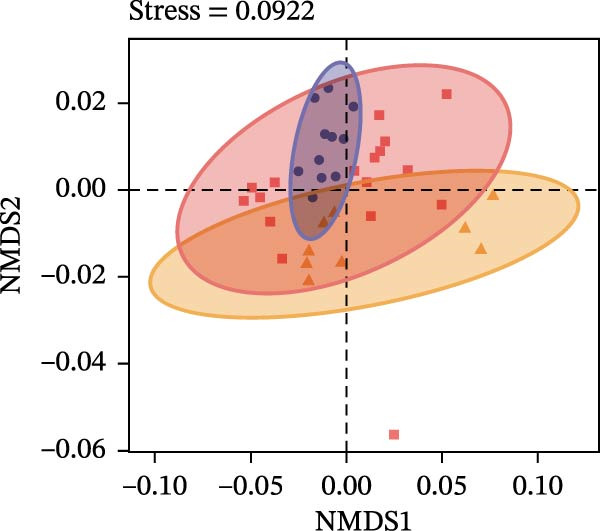
(l)

**Table 1:** Permutational analysis of variance for bacterial taxa composition across four roundworm nematodes.

Factor	Df	Sums of squares	Mean sqs.	F‐model	*R* ^2^	Pr (>F)
Species	1	0.00623	0.00623	3.715	0.069	0.003 ^∗∗^
Genus	2	0.02333	0.01166	6.954	0.256	0.00001 ^∗∗∗^
Sex	1	0.00608	0.00608	3.627	0.067	0.008 ^∗∗^
Residuals	33	0.05535	0.00168	—	0.608	—
Total	37	0.09099	—	—	1	—

*Note*: Df degrees of freedom, F‐model pseudo‐F‐test statistic, *R* ^2^ variance explained and *p*‐value based on 999 permutations. Variance was partitioned using a nested model (~Genus/Species + Sex), where “Species” was nested within “Genus” to account for the hierarchical taxonomic structure of the roundworms.

Significance codes:  ^∗∗^ *p* < 0.01,  ^∗∗∗^ *p* < 0.001.

We further tested for homogeneity of dispersions to distinguish between location and dispersion effects (Table 2 and Supporting Information 2: Tables S1–S3). At the species level, the dispersion was homogeneous (*p* = 0.144), indicating that differences primarily arose from distinct community centroids (compositional shifts) rather than variability (Supporting Information 2: Table S1). However, at the host diet level, omnivores showed significantly lower dispersion than carnivores or herbivores (*p* = 0.006), suggesting that diet may influence the stability rather than the core composition of the microbiome. Overall, the data indicated that the roundworm gut microbiome was structurally constrained by parasite phylogeny, with host diet and worm sex acting as secondary modulators.

**Table 2:** Tests for homogeneity of group dispersions (betadisper analysis).

Factor	F‐statistic	Pr (>F)
Species	1.869	0.144
Genus	4.138	0.023 ^∗^
Sex	2.905	0.117
Host diet	5.778	0.006 ^∗∗^

*Note*: F‐statistic based on ANOVA test; *p*‐value based on 999 permutations.

Significance codes:  ^∗^ *p* < 0.05,  ^∗∗^ *p* < 0.01.

### 3.3. Phylogeny‐Driven Taxonomic Signatures

Given that parasite identity drove community structure, we examined the specific taxonomic signatures distinguishing these lineages. Stacked bar plots showed that all four roundworm species were dominated by the same high‐abundance OTUs (e.g., *Escherichia*, *Salmonella*, and *Klebsiella*), but boxplots revealed notable, statistically supported differences in their relative contributions (Figure 3a,b). In Bs, *E. coli* and *Escherichia fergusonii* were more abundant, while As showed relative enrichment of *S. enterica* and a distinct profile for other Enterobacteriaceae compared with Bs. The two *Toxocara* species (Tc and Tv) exhibited highly congruent abundance profiles, co‐enriched in *Leclercia* sp. W17, *K. pneumoniae*, and *E. marmotae*. Pairwise Wilcoxon tests with Holm–Bonferroni correction confirmed that many of these species‐level differences were statistically significant (*p*_adj < 0.05), particularly when adding inter‐genus comparisons (e.g., Bs vs. As and Bs vs. Tc/Tv). In contrast, when communities were grouped by worm sex or host diet, significantly different taxa were fewer and exhibited smaller effect sizes, in line with the weaker separation seen in the NMDS ordinations. In addition, within inter‐genus comparisons, systematic shifts in the relative abundances of shared core taxa emerged as the main features distinguishing lineages: for example, Bs tended toward higher relative abundances of *E. coli* and *S. enterica*, As were more enriched in *S. flexneri* and *Enterobacter cloacae*, and Tc and Tv shared a closely matched profile. Thus, despite extensive overlap in the “membership list” of high‐abundance OTUs, reproducible and lineage‐specific differences in their relative abundances constituted the principal compositional signatures distinguishing roundworm species and genera.

Figure 3Shifts in the relative abundance of core Enterobacteriaceae species distinguish roundworm lineages. (a) Stacked bar charts display the mean relative abundance of the major bacterial species, stratified by roundworm species, genus, sex, and host diet. (b) Box plots quantify the significant abundance differences of the top five dominant species (*E. coli*, *S. enterica*, *E. marmotae*, *K. pneumoniae*, and *E. fergusonii*) across the different groups. While the core membership is conserved, the abundance proportions shift significantly between groups. Statistical significance was determined by the Kruskal–Wallis test followed by pairwise Wilcoxon rank‐sum test ( ^∗^ *p* < 0.05,  ^∗∗^ *p* < 0.01).

**Figure:**
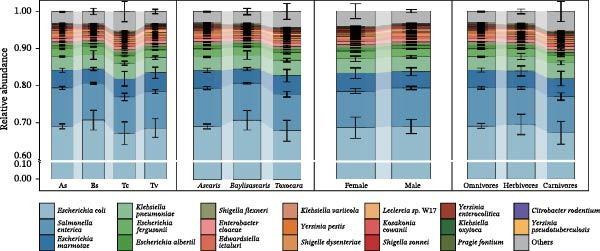
(a)

**Figure:**
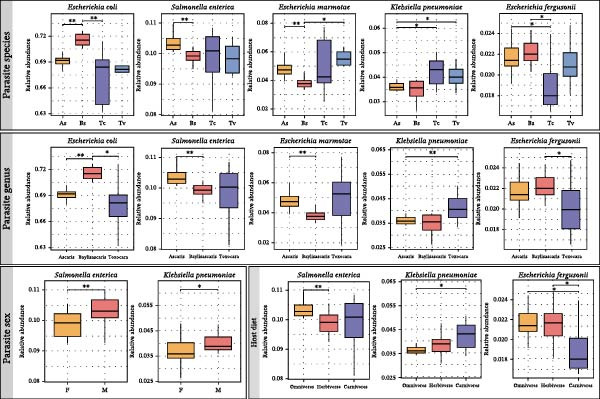
(b)

To further dissect the relative contributions, we performed hierarchical clustering based on both the full OTU dataset and the top 20 most abundant species (Figure 4a). The heatmap of the top 20 species revealed clear, species‐specific abundance signatures, and hierarchical clustering based on the top 20 dominant species also recapitulated the evolutionary history of the four roundworm parasites (Figure 4b). It was noteworthy that Tc and Tv clustered together first, and then grouped with As, while Bs formed the most distant branch. This clustering perfectly mirrored the confirmed phylogenetic relationships within the order Ascaridida [32, 33]. Crucially, a parallel clustering analysis based on host phylogeny failed to explain the parasite microbiome patterns (e.g., the microbiome of Tc [host: Carnivora] clustered with Tv [host: Artiodactyla] rather than Bs [host: Carnivora]). This discordance provided robust evidence that the parasite’s own evolutionary history, rather than the host’s phylogenetic background, was the primary constraint organizing the nematode gut ecosystem.

Figure 4Hierarchical clustering of the gut microbiota mirrors parasite phylogeny (Phylosymbiosis). (a) Heatmap of all detected OTUs (standardized relative abundance) with hierarchical clustering (left dendrogram). The four roundworm species cluster into distinct groups based on their total microbiome composition. (b) Heatmap of the top 20 dominant species compared against evolutionary trees. The dendrogram on the left clusters the samples based on microbiome composition. The diagrams on the right depict the known phylogenetic trees of the parasites (Left) versus their hosts (Right). The clustering of the microbiome mirrors the parasite phylogeny (e.g., *Tc* and *Tv* clustering together) rather than the host phylogeny (where Carnivores *Tc* and *Bs* would be expected to cluster), supporting the hypothesis of parasite‐driven assembly.

**Figure:**
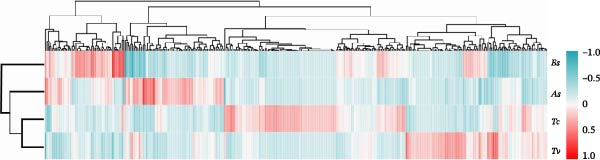
(a)

**Figure:**
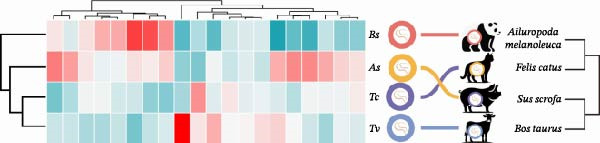
(b)

### 3.4. Functional Potentials and Adaptive Convergence

To understand the biological implications of these structural differences, we predicted the functional profiles of the roundworm gut microbiota using the PICRUSt2 analysis platform (Figure 5). All predicted functions were dominated by core metabolic categories, including basic metabolism, amino acid metabolism, and carbohydrate metabolism, supporting a central role for the microbiota in maintaining the parasite’s survival. Within core metabolisms, the co‐occurrence network topology revealed that metabolic functionality was highly centralized around the dominant Enterobacteriaceae. Key pathways (e.g., carbohydrate metabolism and vitamin metabolism) showed dense positive correlations (Spearman’s *ρ* > 0.6, *p* < 0.05; Figure 5a) with core taxa like *E. coli* and *S. enterica*. This suggested that these core bacteria drove the essential metabolic output required for parasite survival in the hypoxic gut lumen. Despite the shared core, distinct functional biomarkers were identified for each species (LDA score >2.0; Figure 5b). For instance, As was enriched in “xenobiotics biodegradation” and “replication and repair,” potentially pointing to mechanisms for metabolizing anthelmintic drugs. Bs showed enrichment in “carbohydrate metabolism,” likely either an adaptation to the high‐cellulose bamboo diet of the giant panda or a direct response to the giant panda‐specific physiological constraints. Tc was enriched in “cofactor and vitamin metabolism,” whereas Tv showed significantly higher predicted abundance of pathways associated with enhanced ABC transporter, often associated with drug efflux and resistance as reported recently [34, 35]. Interestingly, hierarchical clustering based on predicted functional potential revealed a pattern of convergent evolution. As shown in Figure 5c, phylogenetically distant species Tv and Bs clustered together functionally, diverging from the strict phylogenetic grouping seen in the taxonomic data. This suggested that while the phylogeny constrains the *structure* (who is there), ecological pressures—such as similar nutrient availability or host immune pressures—may drive convergent functional adaptations (what they do) across different roundworm lineages.

Figure 5Functional prediction reveals species‐specific metabolic adaptations centered on an Enterobacteriaceae hub. Functional profiles were predicted from 16S rRNA data using PICRUSt2 (KEGG Level 2). (a) Co‐occurrence network showing significant correlations (Spearman’s *ρ* > 0.6, *p* < 0.05) between specific bacterial species (black‐outlined filled circles) and metabolic pathways (filled circles). The network highlights that core Enterobacteriaceae taxa (large nodes) act as hubs linked to essential metabolic functions. Red lines = positive correlation; Cyan lines = negative. (b) Linear Discriminant Analysis Effect Size (LEfSe) analysis identifying functional pathways significantly enriched (LDA score > 2.0) in each roundworm species (e.g., carbohydrate metabolism in *B. schroederi*; xenobiotics metabolism in *A. suum*). (c) Heatmap of differentially abundant pathways. The top dendrogram shows functional clustering, revealing that *T. vitulorum* and *B. schroederi* share convergent functional profiles despite their phylogenetic distance, suggesting adaptive responses to similar ecological pressures.

**Figure:**
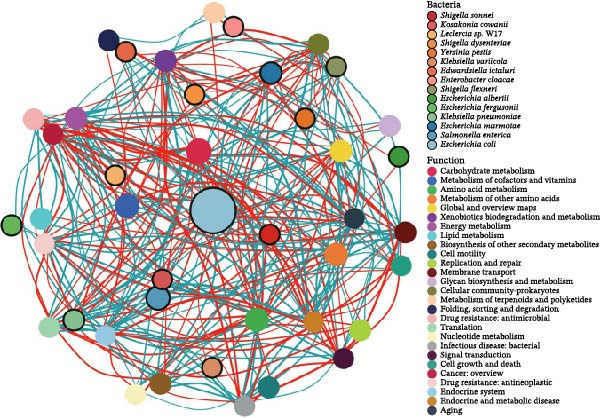
(a)

**Figure:**
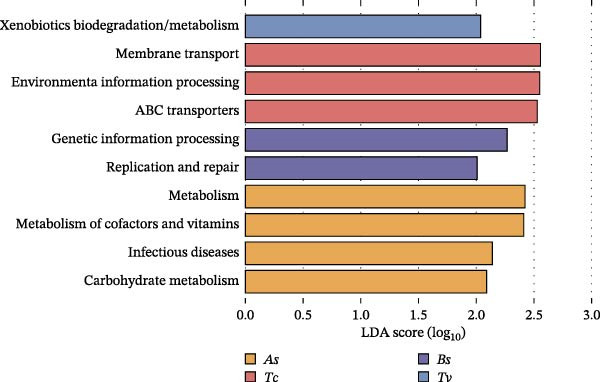
(b)

**Figure:**
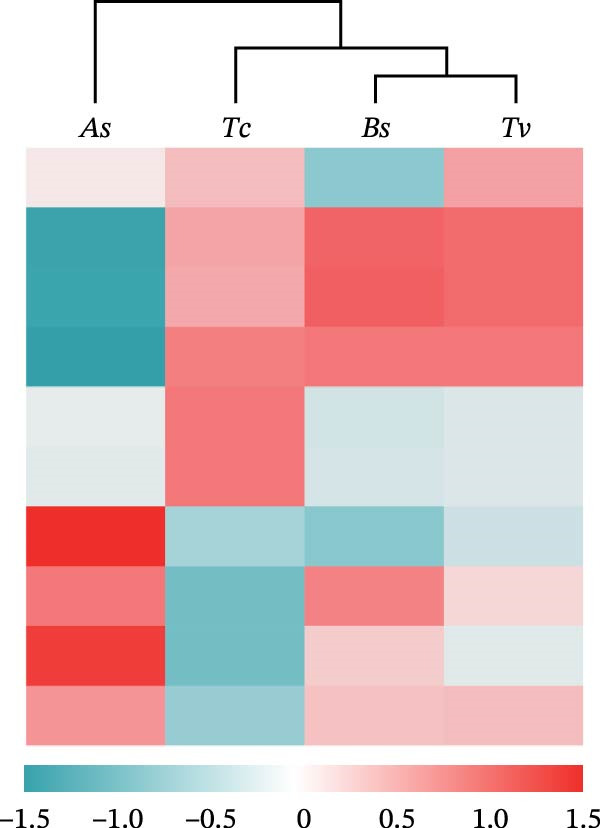
(c)

## 4. Discussion

This study provided the first systematic, cross‐species comparison of gut microbiomes from four major parasitic roundworms—As, Bs, Tc and Tv. Using full‐length 16S rRNA sequencing, we discovered that these parasites harbored a remarkably streamlined bacterial community dominated by the family Enterobacteriaceae. This pronounced dominance suggests that the roundworm intestine may act not as a passive receptacle for the mammalian host’s microbiota, but as an extremely selective “miniature ecological filtration system.” Unlike the complex, stratified ecosystem of the mammalian gut, the roundworm lumen is a closed, nutrient‐limited compartment characterized by low oxygen tension and specific antimicrobial pressures [36]. These conditions create a rigorous ecological filter that excludes the majority of the host’s obligate anaerobes, instead favoring facultative anaerobes with flexible metabolic capacities—such as *Escherichia coli*, *Salmonella enterica*, and *Klebsiella pneumoniae*. These taxa can efficiently exploit simple carbon sources derived from partially digested host material and the parasite’s own secretions [37], explaining the “island ecosystem” pattern observed here: low diversity, high dominance, and high mutualistic compatibility. Interestingly, this pronounced dominance of Enterobacteriaceae has also been described as specific symbioses in entomopathogenic nematodes and insects [38, 39]. It is reasonable to assume that such a high dominance of certain gut bacteria may optimize metabolic efficiency in the nutrient‐limited parasite gut. However, the low microbial diversity could also render the parasite “holobiont” vulnerable to external disturbances, such as anthelmintic treatment or host physiological shifts. In addition, the rare biosphere was observed in roundworms, which may serve as a crucial compensatory mechanism. As mentioned by Lynch and Neufeld [40], these low‐abundance taxa could represent a flexible genetic reservoir, providing the functional redundancy necessary for the microbiome to respond to environmental fluctuations or immune pressures, thereby ensuring the long‐term fitness and adaptive capacity of the parasite. Moreover, these low‐abundance populations could also function as an ecological “seed bank” [41], maintaining a state of dormancy or low metabolic activity until triggered by specific environmental cues. Within roundworms, for instance, if the dominant Enterobacteriaceae of the parasites are suppressed by a host‐derived antimicrobial peptide or a specific drug, distinct members of the rare biosphere with compatible metabolic functions could proliferate to fill the vacated niche. This aligns with the concept of “conditionally rare taxa” that disproportionately contribute to community dynamics during environmental perturbations [42]. Thus, the observed rare biosphere likely reflects a trade‐off between metabolic efficiency maintained by the core taxa under stable conditions and ecological resilience provided by rare taxa under stress in the roundworm lineages.

A central finding of this work is the strong phylogenetic signal observed in the microbiome structure. Our beta‐diversity analyses demonstrated that microbial community clustering mirrored the phylogenetic relationships of the parasites (phylosymbiosis) rather than the phylogeny or diet of the mammalian hosts. For instance, the microbiomes of Tc (from carnivores) and Tv (from herbivores) were more similar to each other than to other parasites inhabiting similar hosts. This supports the hypothesis that the parasite’s own evolutionary history is the primary determinant of community assembly [43].

This “phylogenetic filtering” likely stems from the conserved morphology, physiology, and immune milieu of the roundworm intestine, which impose stable, lineage‐specific selective pressures on resident microbes over evolutionary time. This mirrors patterns observed in vertebrate evolution, where host phylogeny often supersedes environmental factors in shaping microbial composition [44]. Consequently, our data suggested a co‐evolutionary process where the parasite provides a consistent ecological niche, and the microbiota contributes metabolic functions that enhance parasite fitness.

Besides, although the taxonomic structure was constrained by phylogeny, our functional predictions revealed that metabolic potential was shaped by immediate ecological demands, illustrating an “incomplete coupling of taxonomic and functional diversity” [45]. The core “membership” (Enterobacteriaceae) was consistent among different parasites, but different species exhibited specific functional enrichments that appear adaptive. It is well‐known that Enterobacteriaceae is a prominent component of the human gut microbiome and has been implicated in gastrointestinal symptoms such as vomiting and diarrhea [46]. However, its role in the biology and fitness of parasitic roundworms needs further clarification. Regarding dietary adaptation, *B. schroederi* showed significant enrichment in carbohydrate metabolism pathways [47]. Although its host (giant panda) is phylogenetically a carnivore, the giant panda’s high‐fiber bamboo diet likely creates a specific nutrient milieu that the parasite’s microbiome has adapted to exploit. Of course, we cannot rule out that the high abundance of carbohydrate‐metabolizing bacteria (e.g., Enterobacteriaceae) was partially driven by the available bamboo‐derived substrates in the giant panda gut. This aligns with Groussin et al. [48], who proposed that while phylogeny shapes lineage divergence, diet equally shapes ancient metabolic functions. For xenobiotic metabolism, *A. suum* was enriched in pathways for xenobiotic biodegradation. Given that commercial pigs are frequently exposed to anthelmintics and antibiotics, this enrichment may reflect a microbiome‐mediated mechanism for drug detoxification or resistance. Notably, the functional clustering did not strictly follow the phylogenetic tree; Tv and Bs clustered together functionally despite being phylogenetically distant. This functional convergence suggests that different bacterial combinations can perform similar metabolic roles (e.g., redox balance and energy production) in response to similar environmental pressures, such as the hypoxic conditions of the parasite gut.

Furthermore, this study established that roundworm nematodes possess a distinct, taxonomically constrained, and functionally differentiated gut microbiome. The assembly of this community was primarily driven by the parasite’s evolutionary history (“who you are”) but was fine‐tuned functionally by ecological context (“where you live”). These findings challenge the traditional view of parasites as merely pathogens, highlighting them as autonomous hosts regulating complex symbiotic networks. Understanding this “host‐–parasite–microbe” tripartite interaction would offer new conceptual avenues for studying nematode biology and potential microbiome‐targeted interventions for parasite control.

Several limitations of this study warrant consideration. First, the use of 16S rRNA gene sequencing, while robust for taxonomy, provided only predicted rather than actual functional profiles. Future multi‐omics approaches (metagenomics and metabolomics) are required to definitively identify active metabolic pathways and specific parasite–microbe interactions. Second, our cross‐sectional design only captured a single time point; it remains unclear how the microbiome changes across the parasite’s lifecycle, particularly during tissue‐migrating larval stages [25]. Third, given that the parasites were collected from their natural hosts in diverse environments, the study design cannot fully decouple parasite phylogeny from host‐specific factors. While our statistical analyses pointed to phylogeny as a primary driver, alternative explanations for the observed microbiome distinctiveness, such as differences in host diet, gut physiology, or immune status, cannot be definitively excluded. Future studies using controlled infections of different roundworm species within a single permissive host model would be valuable to definitively disentangle the effects of parasite phylogeny from host environmental variables. Finally, although we hypothesize that the microbiome contributes to parasite fitness (e.g., via drug degradation or nutrient synthesis), experimental validation in gnotobiotic or ex vivo systems is necessary to establish causality.

## 5. Conclusions

**Figure 6:**
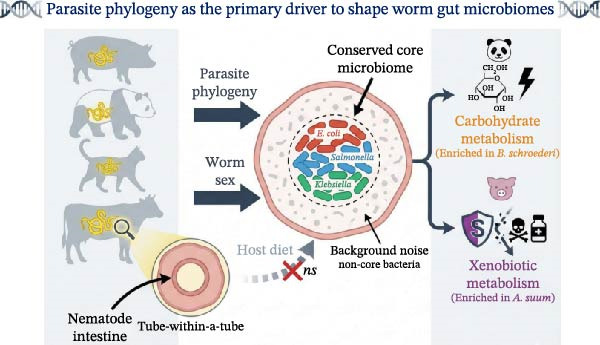
Parasite phylogeny is the primary driver of microbial community structure, followed by worm sex and host diet. A conserved core microbiome (e.g., *E. coli*, *Salmonella*, *Klebsiella*) is shared across species, while non‐core bacteria support species‐specific metabolic specialization—carbohydrate metabolism in *B. schroederi* and xenobiotic metabolism in *A. suum*.

In conclusion, this study provided the first systematic profiling of roundworm gut microbiomes using full‐length 16S rRNA sequencing, revealing that these parasites maintain a remarkably streamlined, Enterobacteriaceae‐dominated ecosystem largely independent of their mammalian hosts. Our data demonstrated that the community assembly is primarily constrained by parasite phylogeny rather than host diet or worm sex, establishing the parasite as an autonomous host that exerts strong evolutionary selection on its internal microbiota (Figure 6). Crucially, while the taxonomic core is conserved, we identified distinct functional signatures tailored to specific ecological niches—ranging from enhanced carbohydrate metabolism in the giant panda parasite Bs to xenobiotic biodegradation in the swine parasite As—suggesting that these bacterial symbionts actively contribute to parasite metabolic flexibility and potential drug resistance. These findings redefine the roundworm as a complex holobiont and underscore the parasite microbiome as a promising, untapped target for therapeutic intervention, paving the way for future multi‐omics research to unravel the mechanistic basis of this tripartite symbiosis.

## Author Contributions

Xinyi Fan, Lei Deng, and Yue Xie conceived and designed the study. Xinyi Fan, Xuan Zhou, and Lidan Wang performed the experiments and data curation. Xinyi Fan, Xinhui Zhang, Yuanyuan Shen, and Yating Xiao drafted the original manuscript and conducted visualization. Hui Wang, Lei Deng, and Yue Xie developed the methodology and performed the review and editing. Yue Xie conceptualized the study and acquired funding.

## Funding

This work was supported by a grant from the Key Project of Sichuan Science and Technology Education Joint Fund (Grant 2025NSFSC2026), the National Natural Science Foundation of China (Grants 32273028, 32503056, and 32573388), the National/Provincial Undergraduate Training Program on Innovation and Entrepreneurship (Grant 202510626001), and the Shanghai Pujiang Talent Program (Grant 25PJA159).

## Conflicts of Interest

The authors declare no conflicts of interest.

## Supporting Information

Additional supporting information can be found online in the Supporting Information section.

## Supporting information


**Supporting Information 1** Figure S1: Rarefaction analysis and species abundance distribution. (a) Shannon rarefaction curves for all 38 individual roundworm samples reach a clear plateau, confirming that the sequencing depth was sufficient to capture total microbial diversity. (b) Rank‐abundance curves exhibiting a steep decline and a distinct “long tail” of low‐abundance taxa. This distribution characterizes the community structure as having few dominant core species and a diverse rare biosphere, which may represent a flexible genetic reservoir for environmental adaptation within the parasite gut.

**Supporting Information 2** Table S1 : Pairwise comparisons of group dispersions among roundworm species (betadisper with Tukey HSD). Table S2: Pairwise comparisons of group dispersions among roundworm genera (betadisper with Tukey HSD). Table S3: Pairwise comparisons of group dispersions among host diet groups (betadisper with Tukey HSD).

## Data Availability

The 16S rRNA sequencing data from this study are publicly available in the NCBI Sequence Read Archive (SRA) under BioProject Accession Number PRJNA1344264.
