# Supplementary figures and images for: Comparative Analysis of Gut Microbiomes in Parasitic Roundworms Reveals Phylogeny‐Associated Community Structure and Functional Adaptation
Source: Transbound Emerg Dis. 2026 Apr 30;2026:2764696. doi: 10.1155/tbed/2764696 (PMC13129500; doi:10.1155/tbed/2764696)

**a**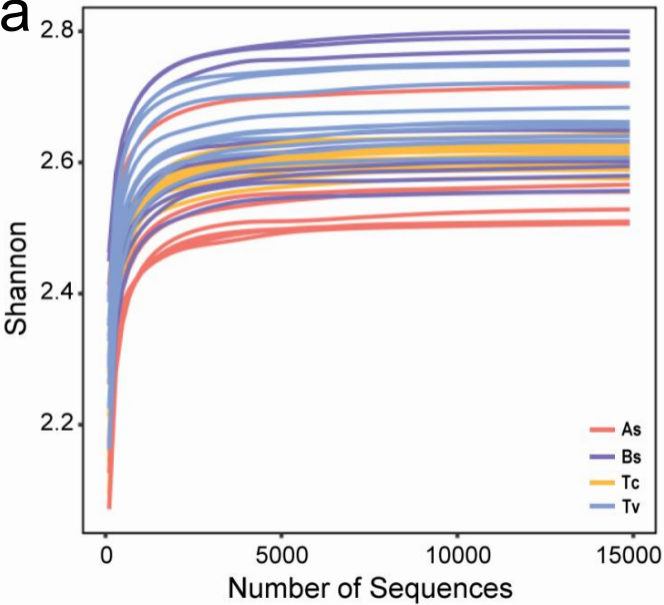**b**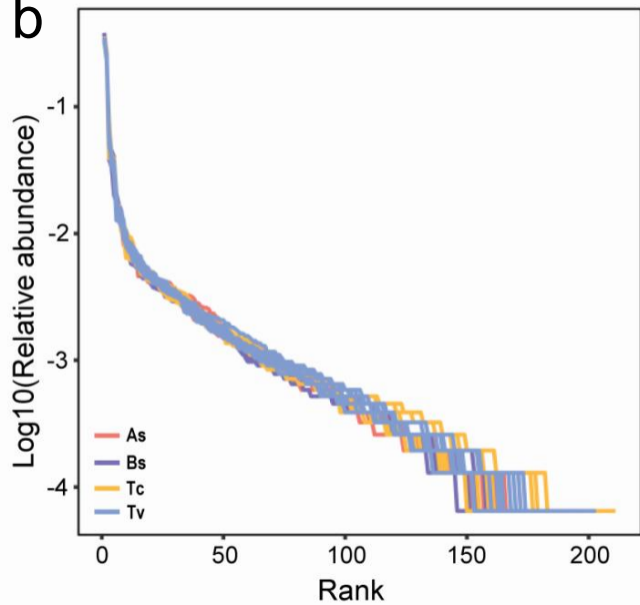

Supplement: Supplementary file 1 — Supporting Information 1 Figure S1: Rarefaction analysis and species abundance distribution. (a) Shannon rarefaction curves for all 38 individual roundworm samples reach a clear plateau, confirming that the sequencing depth was sufficient to capture total microbial diversity. (b) Rank‐abundance curves exhibiting a steep decline and a distinct “long tail” of low‐abundance taxa. This distribution characterizes the community structure as having few dominant core species and a diverse rare biosphere, which may represent a flexible genetic reservoir for environmental adaptation within the parasite gut. [file TBED-2026-2764696-s001.pdf]
