# Supplementary material for: Comparative Analysis of Gut Microbiomes in Parasitic Roundworms Reveals Phylogeny‐Associated Community Structure and Functional Adaptation
Source: Transbound Emerg Dis. 2026 Apr 30;2026:2764696. doi: 10.1155/tbed/2764696 (PMC13129500; doi:10.1155/tbed/2764696)
Supplement: Supplementary file 2 — Supporting Information 2 Table S1 : Pairwise comparisons of group dispersions among roundworm species (betadisper with Tukey HSD). Table S2: Pairwise comparisons of group dispersions among roundworm genera (betadisper with Tukey HSD). Table S3: Pairwise comparisons of group dispersions among host diet groups (betadisper with Tukey HSD). [file TBED-2026-2764696-s002.docx]

**Supplementary Table S1 Pairwise comparisons of group dispersions among roundworm species (betadisper with Tukey HSD)**

| Comparison | Difference | 95% CI lower | 95% CI upper | P_adj |
| --- | --- | --- | --- | --- |
| Bs-As | 0.00628 | -0.01248 | 0.02504 | 0.803 |
| Tc-As | 0.01483 | -0.00256 | 0.03222 | 0.117 |
| Tv-As | 0.00337 | -0.01352 | 0.02025 | 0.949 |
| Tc-Bs | 0.00855 | -0.01133 | 0.02843 | 0.655 |
| Tv-Bs | -0.00291 | -0.02235 | 0.01652 | 0.977 |
| Tv-Tc | -0.01146 | -0.02958 | 0.00666 | 0.335 |

As = *Ascaris suum*; Bs = *Baylisascaris schroederi*; Tc = *Toxocara cani*s; Tv = *Toxocara vitulorum*

Difference = difference in average distance to centroid between groups; CI = confidence interval; *p* adj = *p*-value adjusted using Tukey's HSD method. Significance codes: * p < 0.05, **p < 0.01.

**Supplementary Table S2 Pairwise comparisons of group dispersions among roundworm genera (betadisper with Tukey HSD)**

| Comparison | Difference | 95% CI lower | 95% CI upper | P_adj |
| --- | --- | --- | --- | --- |
| *Baylisascaris*-*Ascaris* | 0.00628 | -0.00842 | 0.02098 | 0.554 |
| *Toxocara*-*Ascaris* | 0.01329 | 0.0019 | 0.02469 | 0.019* |
| *Toxocara*-*Baylisascaris* | 0.00701 | -0.00665 | 0.02068 | 0.429 |

|  |
| --- |

Difference = difference in average distance to centroid between groups; CI = confidence interval; *p* adj = *P*-value adjusted using Tukey's HSD method. Significance codes: * p < 0.05, **p < 0.01.

**Supplementary Table S3 Pairwise comparisons of group dispersions among host diet groups (betadisper with Tukey HSD)**

| Comparison | Difference | 95% CI lower | 95% CI upper | P_adj |
| --- | --- | --- | --- | --- |
| Herbivores-Carnivores | 0.00165 | -0.01193 | 0.01523 | 0.952 |
| Omnivores-Carnivores | -0.01483 | -0.02936 | -0.0003 | 0.044* |
| Omnivores-Herbivores | -0.01648 | -0.0289 | -0.00406 | 0.007** |

Note: Difference = difference in average distance to centroid between groups; CI = confidence interval; *p* adj = *p*-value adjusted using Tukey's HSD method. Significance codes: * p < 0.05, **p < 0.01.
